# APOBEC3G mRNA expression in exposed seronegative and early stage HIV infected individuals decreases with removal of exposure and with disease progression

**DOI:** 10.1186/1742-4690-6-23

**Published:** 2009-03-02

**Authors:** Joel A Vázquez-Pérez, Christopher E Ormsby, Ramón Hernández-Juan, Klintsy J Torres, Gustavo Reyes-Terán

**Affiliations:** 1 Centro de Investigación en Enfermedades Infecciosas, Instituto Nacional de Enfermedades Respiratorias, México City, México

## Abstract

**Background:**

APOBEC3G is an antiretroviral factor that acts by inducing G to A mutations. In this study, we examined the expression of APOBEC3G in uninfected HIV-1 exposed individuals at the time of their partner's diagnosis and one year later. We then compared this expression with that of infected individuals at different disease stages. APOBEC3G mRNA was measured in PBMCs from three groups: healthy controls with no known risk factor to HIV infection (n = 26), exposed uninfected individuals who had unprotected sex with their HIV+ partners for at least 3 months (n = 37), and HIV infected patients at various disease stages (n = 45), including 8 patients with low HIV viral loads < 10,000 copies/mL (LVL) for at least 3 years. Additionally, we obtained sequences from the env, gag, pol, nef, vif and the LTR of the patients' virus.

**Results:**

Exposed uninfected individuals expressed higher APOBEC3G than healthy controls (3.86 vs. 1.69 relative expression units), and their expression significantly decreased after a year from the HIV diagnosis and subsequent treatment of their partners. Infected individuals showed a positive correlation (Rho = 0.57, p = 0.00006) of APOBEC3G expression with CD4+ T cell count, and a negative correlation with HIV viremia (Rho = -0.54, p = 0.00004). The percentage of G to A mutations had a positive correlation (Rho = 0.43, p = 0.0226) with APOBEC3G expression, and it was higher in LVL individuals than in the other patients (IQR 8.27 to 9.64 vs. 7.06 to 8.1, p = 0.0084). Out of 8 LVLs, 3 had hypermutations, and 4 had premature stop codons only in viral *vif*.

**Conclusion:**

The results suggest that exposure to HIV may trigger APOBEC3G expression in PBMCs, in the absence of infection. Additionally, cessation of exposure or advanced disease is associated with decreased APOBEC3G expression.

## Background

Human APOBEC3G (hA3G) is a cellular antiretroviral factor with a potent inhibitory effect on HIV replication. The cytidine deaminase activity of hA3G catalyses the conversion of cytosine to uracil on the negative-strand viral cDNA[1]. In vitro, hA3G inhibits HIV replication by causing G to A hypermutations, preferentially in the GG dinucleotide context. These mutations often alter the amino acid sequences of multiple viral gene products and introduce lethal stop codons, thereby compromising HIV replication [2-4]. In contrast, APOBEC3F acts preferably in the GA context. In addition to G to A hypermutation, other h3AG antiretroviral mechanisms have been proposed, which include interaction with the HIV nucleocapsid with subsequent inhibition of tRNA^Lys3 ^annealing to viral RNA, and DNA strand transfer during reverse transcription [2,3]. Recently, studies in murine models suggest a link between hA3G and virus-specific neutralizing antibody responses in Friend Virus (FV) infection[4]. As a countermeasure to hA3G restriction, HIV-1 neutralizes the antiretroviral activity of hA3G by inducing its ubiquitination and degradation through the Vif protein [5-7]. In vivo, the significance of hA3G-induced hypermutation as a protecting agent of clinical pathogenesis and HIV disease progression remains uncertain. One study suggested that hA3G mRNA levels in activated PBMCs correlated negatively with plasma HIV RNA levels and positively with CD4+ T cell counts[8]. In contrast, a different research group did not find any correlation[9]. On the other hand, one report showed significantly increased hA3G mRNA in PBMCs and cervical biopsy cells from HIV-exposed seronegative individuals (ES)[10], and a separate report found that stimulated PBMCs of long term non-progressors (LNTP) had significantly higher hA3G mRNA levels than either uninfected controls or individuals with progressive HIV disease[8]. Finally, Ulenga et al[11] found that patients with a low viral set point had a higher hA3G expression than patients with high viral set point. Taken together, these studies point to an in vivo HIV neutralizing activity of hA3G.

All the above reports were carried out in different patient groups and experimental conditions, which preclude direct comparisons about differential expression levels between groups like LTNP and ES. Additionally, the question remains whether hA3G is constitutively expressed by these protected patients, or whether its expression is the result of exposure to HIV or its gene products. To address this question, we measured hA3G mRNA expression in unstimulated PBMCs from an ES cohort at the time of first diagnosis of their sexual partners, and after one year of antiretroviral treatment of the infected partner, in order to observe if the expression levels varied with the decrease in viremia of their partners, or remained unchanged. The findings would indicate either induced or constitutive hA3G expression, respectively. In parallel, we contrasted these results with hA3G mRNA expression in HIV infected persons at different disease stages and correlated the expression levels with CD4+ counts and HIV viral load. Separately from these patients, we also studied a subgroup of 8 subjects (low viral load group, LVL) that had remained with HIV viral load < 10,000 copies/mL for at least three years, in order to assess if a sustained control of viremia was related to hA3G expression. Additionally, we measured G to A mutation activity on the viral *vif*, *gag*, *pol*, *nef*, *env *and LTR sequences from 20 randomly selected patients. We also included twenty six healthy subjects (HC group), with no known HIV risk factors as a control group for this study.

## Results

### hA3G mRNA expression in ES, HC and HIV infected patients at different stages of disease

**Figure 1:**
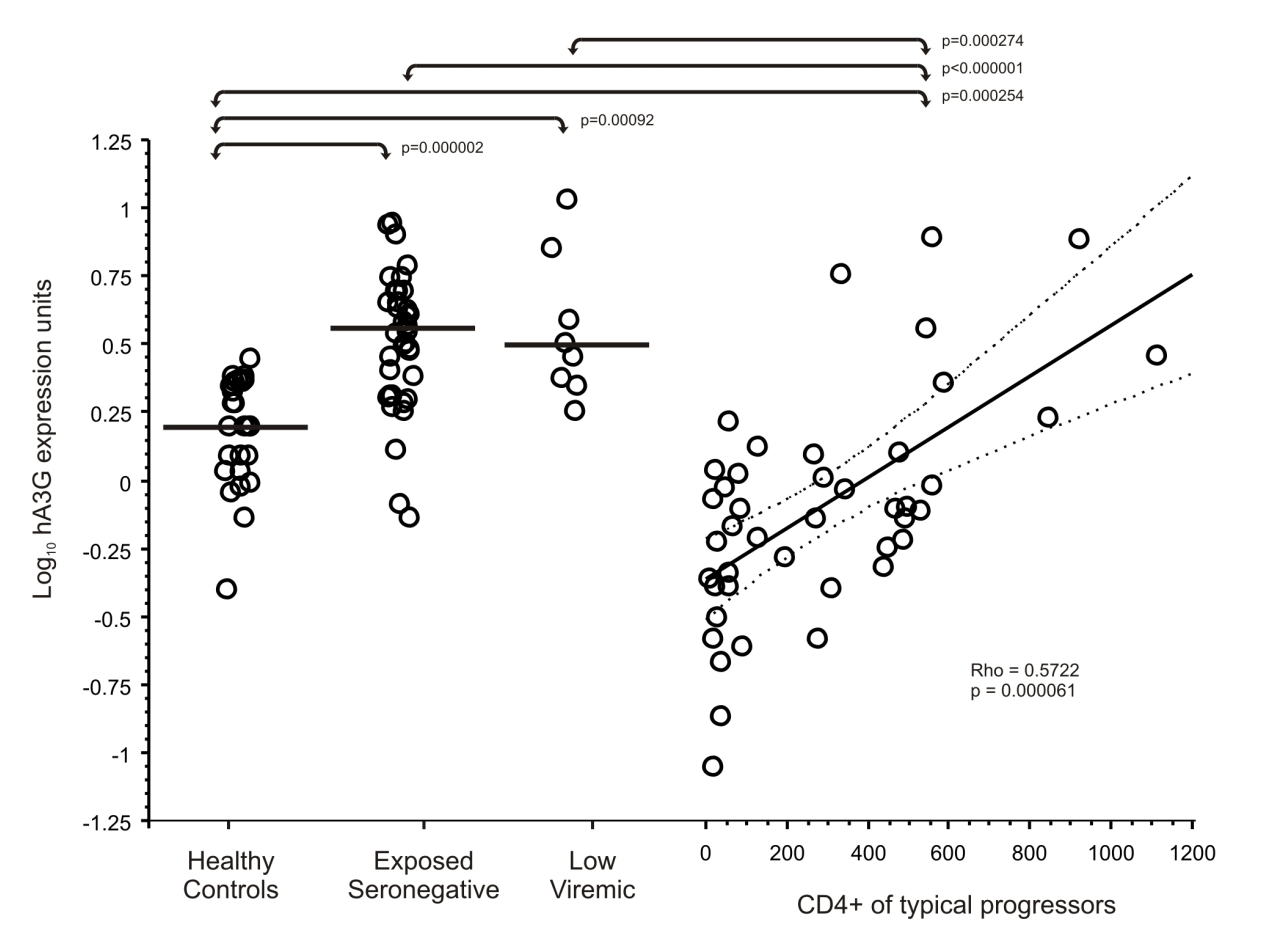
**hA3G mRNA expression in ES, HC and HIV infected patients at different stages of disease**. Results for log hAG3 mRNA expression in healthy control subjects, exposed seronegative individuals, HIV+ patients that have consistently low viral loads (LVL) < 10,000 copies/μL for at least 3 years, and typical progressors. Typical progressors are shown as a regression over CD4+ cell/μL, with Spearman's Rho and significance marked. Horizontal lines show the median, sloped solid line is the linear regression, and the flanking dashed lines show the 95% confidence interval for the mean. Horizontal lines with arrows show the significant differences between groups (Mann-Whitney's U test).

Median hA3G mRNA levels were significantly increased in the PBMCs from ES individuals and LVL individuals as compared to HC and HIV+ patients (Figure 1). We did not find a statistical difference between median hA3G mRNA levels in ES subjects and LVL patients. In HIV+ patients we found a significantly positive correlation between hA3G expression and peripheral blood CD4+ cell count (Figure 1). The overall hA3G mRNA expression was significantly lower in the HIV+ patients than in the HC, ES and LVL individuals (Figure 1), which was expected given the correlations mentioned above and that HIV+ patients had a relatively advanced disease stage (median CD4+ 265, IQR 45.75 to 480.25).

**Figure 2:**
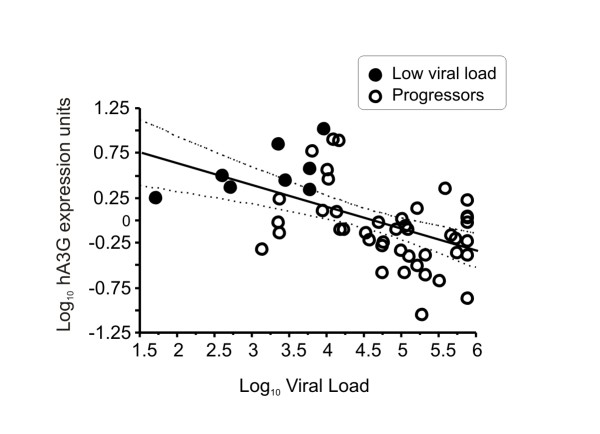
**Relationship between log hA3G mRNA expression with log viral load**. The sloped solid line is the linear regression, and the flanking dashed lines show the 95% confidence interval for the mean (Rho = -0.5418, p = 0.00004). Solid dots represent subjects with persistent < 10,000 copies/mL for more than 3 years, and the open dots are typical progressors.

We found a negative correlation between plasma viral load and hA3G expression (Rho = -0.54, p = 0.00004), as can be seen in Figure 2.

### hA3G mRNA expression in ES after one year of partner's diagnosis

**Figure 3:**
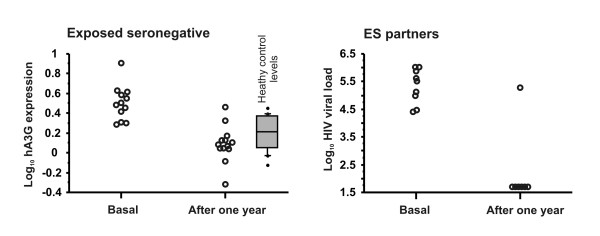
**hA3G mRNA expression in ES after one year ofexposure**. Left panel. hA3G mRNA expression in exposed seronegative subjects at time of diagnosis of their partners, and after one year of follow up. There was a significant decrease (p = 0.0022, Wilcoxon sign test). The inset box plot is healthy control expression level, for reference purposes only. Right panel. Viral load in the partners of the ES subjects after a year of treatment. The overall reduction was also significant (Wicoxon's sign test p = 0.018).

To examine if the high hA3G mRNA expression in ES was constitutive or if it was induced by exposure to HIV antigens, we measured the hA3G mRNA expression in 12 randomly selected ES individuals after one year of diagnosis of their partners (Figure 3, left panel). In all the examined ES individuals, the level of hA3G expression decreased (Wilcoxon sign test p = 0.0022), and reached comparable levels to healthy controls (Figure 3, left panel, box plot inset). In addition, we were able to gather information from some partners of ES individuals that still remained as couples, and collected data on 9 basal samples and 8 samples after a year. Using these samples, we measured the change in their viral loads after a year of antiretroviral treatment. The other 3 ES partners were treated at different institutions, and we did not have access to their data. As can be seen in Figure 3, right panel, all but one infected partner (who reduced from > 750,000 to 194,000) had undetectable HIV levels after a year. The overall reduction was also significant (Wicoxon's sign test p = 0.018).

### Detection of G to A mutation and hypermutation in HIV sequences

**Figure 4:**
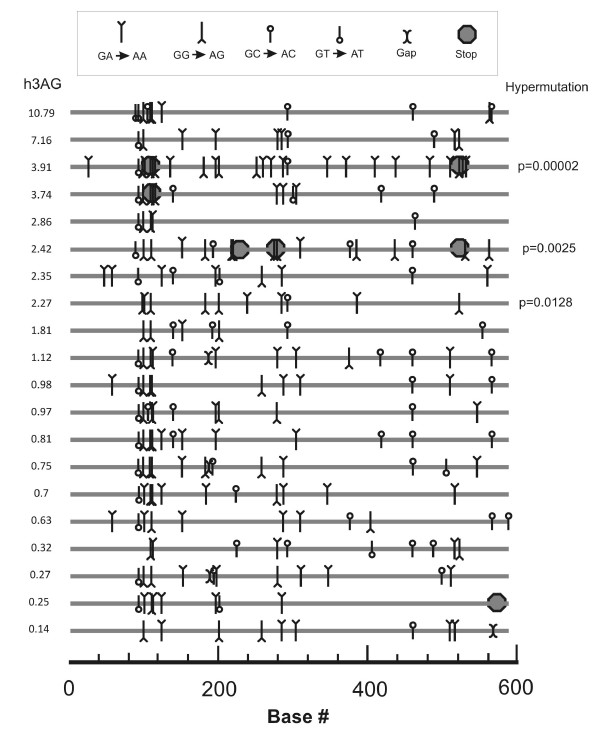
**Analysis of complete *vif *sequences derived from 20 different HIV+ individuals**. Graphical representation of the G to A changes compared with HIV HXB2 reference. The nucleotide context *GG*, *GA*, *GC *or *GT *represents two contiguous bases, with G to A mutations occurring in the first base. hA3G mRNA levels are shown on the left column and significant hA3G-type hypermutations on the right column. The three significantly hypermutataded sequences are from patients having < 10,000 viral copies/mL for over three years.

In order to test if a high expression of hA3G mRNA correlated with an increased anti- HIV activity, we analyzed the number of G to A mutations in the *gag, pol, env, nef, vif *and LTR sequences from 20 randomly selected LVL and HIV+ individuals. LVL patients had a higher percentage of hA3G-type G to A mutations from the total G content in the analyzed sequences than the rest of the infected patients, with a median value of 8.86% vs. 7.9% (IQR 8.27 to 9.64 vs. 7.06 to 8.1, p = 0.0084, Mann-Whitney's U). Additionally, we found a significant correlation between the level of hA3G mRNA expression and the percentage of G to A mutations (Rho = 0.43, p = 0.0226). There were no significant differences in the percentage of G to A mutations between the different viral genes. Direct sequencing of HIV amplicons revealed hypermutations in only 3 LVL HIV sequences, and they resided only in the *vif *region (Figure 4). Two occurred in the *GG*context (characteristic of hA3G activity), and one occurred in the *GA*context (characteristic of hA3F activity). In order to identify probable Vif mutants that selectively failed to efficiently "silence" hA3G enzymes in LVL individuals, we looked for premature stop codons in *vif *sequences from these patients (Figure 4). Three out of eight *vif *sequences in LVL contained one or more premature stop codons in the context of a Trp codon (TGG to TAG). No stop codons were found in *vif *sequences of HIV+ individuals that were not LVL. Motifs SQLYLAL and Y^40^RHHY^44^, which are essential for ubiquitination and interaction with hA3G, respectively [12,13], were totally conserved in all sequences. We did not observe hypermutations or premature stop codons in the *gag, pol, nef, env *or LTR regions of HIV.

## Discussion

The role of hA3G in regulating HIV replication in vivo is unclear. In this study, we demonstrated that individuals exposed to HIV, either through unprotected sex with an infected partner or during a non-AIDS phase of HIV infection, have an increased expression of hA3G mRNA, as shown directly by the expression levels in ES individuals, and by the positive correlation between CD4+ cell count and hA3G mRNA expression. Once the virus overcomes the immune system of the patient, hA3G mRNA expression is almost completely suppressed. This can be seen by the negative correlation between hA3G mRNA expression and HIV plasma viral load, and by the lower hA3G mRNA expression seen in the advanced HIV+ patients.

Further supporting the notion that hA3G mRNA expression is related to exposure to HIV gene products, the viremia levels of the ES's partners were significantly reduced a year after HIV diagnosis and subsequent antiretroviral treatment, coinciding with a reduction in hA3G mRNA expression in the ES subjects. The dramatic reduction in plasma HIV viral load most probably reduced exposure of ES subjects to the viral gene products of their partners. An additional reduction of exposure to HIV gene products would result if the ES individuals adopted safer sex practices, and it is reasonable to assume that at least some of the ES initiated these safer sex practices after being informed of their partner's diagnosis. Even though we cannot fully document a lack of further HIV exposure in our ES cohort, this notion appears to be the best explanation for the reduction in hA3G mRNA expression in the ES individuals.

In this study, we found increased levels of hA3G mRNA in the unstimulated PBMCs of two groups in the same cohort: exposed seronegative and LVL individuals. These increased hA3G mRNA levels are in agreement with previous reports that examined CD3 and CD28-stimulated PBMCs in long term non-progressors[8] and in ES individuals[10]. Here we found that ES and LVL groups have similar values of hA3G mRNA expression, which may suggest that ES individuals regulate hA3G in a similar manner as LVL patients.

The negative correlation of hA3G expression with viremia described in the present study (Figure 2) is in agreement with and clarifies other reports. Jin et al[14] have speculated that HIV infection can induce different discrete stages of hA3G expression, which is in concordance with our findings. Additionally, Ulenga et al. [11] recently reported that patients with a higher viral set point expressed less hA3G and hA3F mRNAs than low viremic set point patients, also suggesting that hA3G expression is suppressed in advanced disease.

Jin et al[14] suggested that the observed discrete stages of hA3G expression arise by naturally selecting from the general population individuals that constitutively express high levels of hA3G. Our data do not support this contention, since the ES individuals decreased their hA3G mRNA expression after a year of their partners' diagnoses and treatment, and return to healthy control levels, which suggested that they had the same constitutive hA3G expression than that of the general population, but that they induced this expression after coming into contact with HIV gene products. However, there is a high degree of variability in the hA3G mRNA expression in the ES individuals, and there are suggestions that expression may be bounded[14], so that the final level of expression is the result of the contributions from host genetic susceptibility and the amount of HIV exposure. It remains to be studied if the ES individuals have a genetic susceptibility to have a higher or faster induced expression to initial HIV exposure, and this is what conferred to them their apparent immunity.

The present methodology cannot definitively assess if the reduction in hA3G mRNA in PBMCs is the result of a larger proportion of hA3G expressing cells or an increase in hA3G expression at the single cell level. However, the fact that ES subjects had similar CD4+, CD8+ T cells and monocyte PBMC composition at the basal and one year measurements (data not shown) suggests that the different expression levels are reflected at the single cell level. Furthermore, it has been established that ES individuals can generate anti-HIV-1 T cell responses while remaining uninfected, but lose these responses after ceasing exposure to HIV[15]. T cells produce interleukin-2 (IL-2) in response to stimulation by synthetic HIV-1 *env*-derived peptides, and Stopak et al. have observed that IL-2 and IL-15 activated *de novo *hA3G gene expression in primary PBMCs[16]. Thus, the secretion of these cytokines could explain the increased levels of hA3G mRNA in ES individuals.

The mutation data obtained from the 20 individuals showed a significant correlation between the percentage of G to A mutation and hA3G mRNA levels and a higher G to A mutation rate in the LVL subjects. Potentially dysfunctional hypermutated regions were found in three sequences, and four sequences had premature stop codons. This incidence of hypermutated sequences did not correlate with hA3G mRNA expression, as was reported elsewhere [17]. This is possibly due to differences in the sample size and the length of the sequences analyzed. Taken together, the data suggest that the expression of hA3G mRNA is associated with the telltale signs of APOBEC3 activity on the viral genome.

Pillai et al. have hypothesized that there is a dose-dependent response between intracellular hA3G concentrations and the degree of viral hypermutation[18]. Also, they assumed that while an effective inhibition of *vif *may result in mutational extinction, weak inhibition may accelerate evolution of drug resistance, and immune escape. Our data do not shed light on ether a mutational extinction or accelerated evolution.

Chiu et al. [19] reported a distinction between the active low molecular weight form (LMW) of hA3G and its inactive high molecular weight form (HMW). In this regard Stopak et al. [16] had demonstrated that activation by cytokine treatment induced a shift of LMM hA3G to its HMM conformation, paralleled by an increase in susceptibility to HIV infection. On the other hand, Vif could also induce conformational changes of hA3G into HMM complexes [20,21]. With the methodology described here, we cannot quantify how much mRNA will be translated into LMM or HMM forms, which could be important for viral control. However, we could observe that APOBEC3-like action on the viral genomes correlated with mRNA expression. This could be due to LMM forms induced in dendritic cells [15].

Recently one report showed that HIV hypermutations correlated with CD4+ counts [22]; however, we did not find this correlation, and we only found an increased G to A mutation rate, without reaching a statistically significant hypermutated score. In conclusion, our study shows that levels of hA3G mRNA are increased in ES individuals and in early stages of HIV infection, while the levels are decreased a year after their partners' diagnoses and treatment, suggesting that hA3G expression is induced by exposure to HIV.

## Methods

### Patients

The study sample consisted of 108 subjects. 26 were healthy controls (HC) free of HIV risk factors, screened with a blood donation questionnaire. 37 were HIV-exposed seronegative individuals (ES) and had a history of multiple unprotected sexual episodes within three months before entering the study. The continued seronegative status was verified for at least one year with subsequent serum samples analyzed by ELISA. Additionally, they were tested for the presence of HIV DNA in PBMCs by *gag *and LTR nested PCR [23]. 45 were infected patients without antiretroviral treatment (HIV+) and were at different disease stages, with a median of 305 CD4+ cells/μL (IQR 53.25 to 535). These included 8 individuals with low viral loads (LVL), < 10,000 HIV-1 RNA copies/mL for at least three years of follow-up and who were naive to antiretroviral therapy. This study was approved by the Bioethics and Research Committee of the National Institute of Respiratory Diseases, Mexico, and all patients and subjects read and signed informed consent letters.

### Relative expression of hA3G mRNA

Peripheral venous blood samples were collected in EDTA tubes; PBMCs were separated by Ficoll density gradient and stored at -80°C. DNA and total RNA was extracted from PBMCs by QIAGEN Blood minikit and RNeasy minikit (Qiagen, Valencia, CA, USA), respectively.

To measure hA3G mRNA expression, cDNA products were first synthesized from the total RNA with random primers using a Transcriptor First Strand cDNA Synthesis Kit (Roche Diagnostics, Mannheim, Germany). cDNA quantification for hA3G and G6PDH (LightMix, for the detection of human G6PDH, Roche) was performed by real time PCR using FastStart DNA Master Hybprobes and LightCycler thermal cycler 2.0 (Roche). Primer and hybprobe design were specific for hA3G (NM 021822), hu ApoB 3G F2 (CAATAATGACATACAGTGAATTT), hu ApoB 3G R2 (CAGGTCTCTGCCTTCCTTAGA), huApoB3GFL (GACATCCCTGGTGGTCCACA-FL) and hu ApoB 3G LC (LC640-GGTGTCCCAGCAGTGCTTAAA-PH). For each sample, the number of copies of hA3G mRNA was divided by the number of copies of G6PDH mRNA, in order to normalize for hA3G mRNA expression in different cell samples. Relative expression units were calculated as expression of hA3G/expression of G6PDH. The results are given as median relative expression units of triplicate assays.

### Statistical analysis

All statistical analyses were carried out with R statistical programming environment[24] version 2.7.1. Statistical differences between groups were assessed with Mann-Whitney U test, correlations were calculated with Spearman's rho, and paired before-after comparisons were carried out with Wilcoxon's signed rank test. hA3G mRNA expression is shown in figures as a logarithm, since it was derived from a ratio, and was therefore log-normal. This transformation did not affect the results of the statistical tests, since they relied on the ranks of the data.

### Determination of hypermutated sequences

We amplified and sequenced 5 different regions of HIV proviral DNA: P2, P7, P1 and P6 (*gag*), protease and RT (*pol*), V3 loop (*env*), and the entire *vif *and *nef *genes. *nef *and the V3 loop were amplified as previously reported[25,26]. Protease and RT sequences were amplified and analyzed by a Viroseq kit (Celera Diagnostics, Alameda, CA, USA). P2, P7, P1 and P6 of *gag *and the entire *vif *gene were amplified by nested PCR. Initial amplification of *gag *was performed using primers p2p7p1p6F outer (5'ATTGGATGACAGAAACCTTGTTGG3') and p2p7p1p6R outer (5'CTTCTAATACTGTATCATCTGCTCC3'), *vif *initial amplification was performed using primers JVPvifF (5'ACAGCAGAGATCCACT3') and JVPvifR2 (5'AGAATTCTTATTATGGCTTCCA 3'). An aliquot (5 μL) of first round PCR product was then used as a template in a second PCR reaction with primers p2p7p1p6F inner (5'GAAGAAATGATGACAGCATGTC3') and p2p7p1p6R inner (5'CATCTGCTCCTGTATCTAATAG3'), and JVPvifF2 (5'TGGAAAGGACCAGCAAAGCT3') and JVPvifR (CTAGGAAAATGTCTAACAGCTT), respectively. We used High Fidelity Polimerase (Platinum Taq DNA Polymerase High Fidelity, Invitrogen, Carlsbad, California, United States) in all PCR reactions.

Nucleotide sequences of PCR products were determined using BigDye Terminator cycle sequencing kit (Applied Biosystems) and ran on an ABI Prism 3100 analyzer sequencer (Applied Biosystems). Nucleotide sequences of PCR products were aligned and analyzed using Clustal X[27] and MEGA 4.0[28] respectively. Analysis of G to A substitution in proviral sequences was performed using HYPERMUT 2.0 with default settings, available from [29]. This tool can distinguish plus-strand hA3G/F hypermutation, by measuring the number of G to A changes in the GG and GA dinucleotide context, respectively, and testing for a significant increase (p < 0.05) from background levels of mutation.

## Competing interests

The authors declare that they have no competing interests.

## Authors' contributions

JAVP, CEO, RHJ and GRT contributed to the study design. JAVP performed the RT-PCR real time assays, sequencing, analysis and interpretation of the data and wrote the manuscript. CEO carried out the statistical analysis and wrote the manuscript. KJT and RHJ provided patients' samples and summary of clinical data. GRT, KJT, CEO and RHJ assisted with manuscript preparation, and helped to edit the manuscript. All authors read and approved the manuscript.
